# Total Body Metabolic Tumor Response in ALK Positive Non-Small Cell Lung Cancer Patients Treated with ALK Inhibition

**DOI:** 10.1371/journal.pone.0149955

**Published:** 2016-05-03

**Authors:** Gerald S. M. A. Kerner, Michel J. B. Koole, Alphons H. H. Bongaerts, Jan Pruim, Harry J. M. Groen, CTMM Air Force Consortium

**Affiliations:** 1 University of Groningen and Department of Pulmonary Diseases, University Medical Center Groningen, Groningen, the Netherlands; 2 University of Groningen and Department of Nuclear Medicine and Molecular Imaging, University Medical Center Groningen, Groningen, the Netherlands; 3 Department of Nuclear Medicine, Tygerberg Hospital, Stellenbosch University, Stellenbosch, South-Africa; Istituto dei tumori Fondazione Pascale, ITALY

## Abstract

**Background:**

In *ALK*-positive advanced NSCLC, crizotinib has a high response rate and effectively increases quality of life and survival. CT measurement of the tumor may insufficiently reflect the actual tumor load changes during targeted therapy with crizotinib. We explored whether ^18^F-FDG PET measured metabolic changes are different from CT based changes and studied the impact of these changes on disease progression.

**Methods:**

^18^F-FDG PET/CT was performed prior to and after 6 weeks of crizotinib treatment. Tumor response on CT was classified with RECIST 1.1, while ^18^F-FDG PET response was assessed according to the 1999 EORTC recommendations and PERCIST criteria. Agreement was assessed using McNemars test. During follow-up, patients received additional PET/CT during crizotinib treatment and second generation *ALK* inhibition. We assessed whether PET was able to detect progression earlier then CT.

**Results:**

In this exploratory study 15 patients were analyzed who were treated with crizotinib. There was a good agreement in the applicability of CT and ^18^F-FDG PET/CT using the EORTC recommendations. During first line crizotinib and subsequent second line *ALK* inhibitors, PET was able to detect progression earlier then CT in 10/22 (45%) events of progression and in the others disease progression was detected simultaneously.

**Conclusion:**

In advanced *ALK* positive NSCLC PET was able to detect progressive disease earlier than with CT in nearly half of the assessments while both imaging tests performed similar in the others.

## Introduction

In clinical practice, tumor response measurements are performed using the anatomical CT based RECIST criteria[1]. Nowadays, it has been recognized that metabolic tumor changes as measured with ^18^F-FDG PET can also be used as an indicator of effectiveness (EORTC recommendations[2], PERCIST[3]). Examples of this principle include imatinib treated gastrointestinal stromal tumors and EGFR-TKI treatment for EGFR mutated advanced NSCLC [4–7]. During targeted therapy, early metabolic changes in tumor activity often precedes anatomic tumor lesion size alterations. *ALK* positive advanced NSCLC are treated with different *ALK* inhibitors such as crizotinib, ceritinib and alectinib[8–11]. Whether targeted treatment such as crizotinib induces quick metabolic changes, and whether these metabolic changes are related to lesions size alterations is currently unknown.

The goal of this paper is to describe metabolic responses on crizotinib in *ALK* positive NSCLC patients and compare PET and CT assessments with different tumor response criteria. Furthermore, we also assessed during follow up with *ALK* inhibition whether PET is able to detect progression of disease at an earlier time point compared to regular CT.

## Material and Methods

### Patients

Patients with advanced *EML4/ALK* positive NSCLC treated with crizotinib were studied with ^18^F-FDG PET/CT at baseline and after six weeks of treatment. During and after treatment patients underwent PET/CT until progression of disease was determined. If they were eligible for additional treatment (local treatment or systemic treatment with a second generation *ALK* inhibitor), PET/CT was repeated to assess tumor response again until disease progression was determined.

### Informed Consent and Ethics

This study was performed using clinical data from previous studies for which informed consent was obtained. For this study all data were anonymized and de-identified prior to analysis. Under the Dutch Law Medical Research Involving Human Subjects Act (WMO), no (additional) informed consent was necessary from the Institutional Review board.

### Pathology

Tumor samples were obtained either by bronchoscopy, transthoracic lung biopsies, or from resections. Samples were examined according to the 2011 IASLC/ATS/ERS NSCLC classification[12]. ALK status was determined by FISH and/or by immunohistochemistry.

For detecting the ALK fusion gene, the Vysis ALK Break Apart FISH probe (Abbott 06N43-020) was used. A score of at least 50 tumor cell in an area on the paraffine coupe was marked by the pathologist and scored by two different observers. For scoring FISH patterns, the criteria were used as described by Thunnissen et al[13].

To detect ALK expression using immunohistochemistry, a fully automated immunohistochemic assay was used on the Ventana BenchMark Ultra with the anti-ALK (D5F3) rabbit monoclonal primary antibody (Ventana Cat. No. 790–4794 / 06679072001). This analysis was performed using the OptiView DAB IHC Detection Kit and the OptiView Amplification Kit. For assessment the Ventana ALK scoring interpretation guide was used (http://www.uclad.com/newsletters/ALK-LUNG-IHC-INTERPRETATION-GUIDE.pdf).

### CT

The diagnostic CT images were made on a Siemens Biograph/Somatom mCT scanner (Siemens Healthcare, Erlangen, Germany). The CT was performed in 8 seconds, (effective mAs 80, 120 kV with care dose setting active) in a craniocaudal direction at full inspiration. Slice thickness was 0.5 mm, pitch was 14 with a rotation of 0.5 seconds. Patients were injected with 55 ml of Iomeron contrast 350 mg/ml (Bracco Imaging Deutschland GmbH, Konstanz, Germany) at a speed of 2.5 ml/sec 30 seconds prior to scanning.

Tumor response was measured on CT according to RECIST 1.1 criteria by an experienced radiologist[1].

### ^18^F-FDG PET/CT

^18^F-FDG PET/CT images were made on the same Siemens Biograph/Somatom mCT time-of-flight scanner according to EANM guidelines.[14, 15] The voxel size of the EANM reconstructions are 4 by 4 by 2.4 mm (38.4 mm^3^). Prior to tracer injection, a blood sample was drawn to confirm fasting blood glucose level (<11 mmol/l) after a 6 hour fasting period. Patients were dosed at 3 MBq/kg bodyweight intravenously. Sixty minutes after injection, patients were scanned from the upper leg to the brain. Scan times per bed position were dependent on patient weight, 1 minute if less than 60 kg, 2 minutes if between 60–90 kg and 3 minutes if above 90 kg per bed position[16].

### ^18^F-FDG PET/ response measurement

All PET based analyses were performed using the IMALYTICS research work station (Philips Technologie Gmbh Innovative Technologies Aachen, Aachen, Germany). Using the maximum intensity projection (MIP), each separate metastasis was visually selected and an adaptive threshold algorithm was used to calculate the volume of interest. The threshold was set to 41% based upon the study by Cheebsumon et al[17]. This was performed with the following settings: 20 mm distance of the background shell from the 70% peak/contour and 2.5 threshold for voxels to be excluded.

1. Two different methods of metabolic response measurement were used. Using the previously defined VOI, five lesions with the highest SUV_max_ were selected, and the SUV_max_ averaged. On the response scan, the same 5 lesions were selected and averaged again. The difference in percentage between these two measurements was used for response. The assessment was performed according to the 1999 EORTC recommendations[2].
2. Tumor response assessment according to the PERCIST criteria[3] was performed separately by using MIM version 6.0.2 (MIM software, Cleveland, OH, USA) to assess the SUV_peak_.

### Follow up

Follow-up was performed in all patients. Patients were assessed at regular times every 6–12 weeks. After disease progression patients received a new treatment and a subsequent progression event was recorded.

### Statistics

All SUV, except for SUV_peak_ in accordance with the PERCIST criteria [3], were corrected for glucose level. The measure of agreement in the applicability between CT response with the EORTC recommendations and PERCIST criteria, respectively, was assessed using the McNemar test. Progression-free survival (PFS) was defined from date of diagnosis until date of tumor progression on CT or death. If a solitary new lesion was detected and was completely treated with a local treatment such as stereotactic radiotherapy, surgery or radiofrequency ablation and no regrowth was determined for at least 3 months, this single event was not considered as progressive disease.

All statistics were performed using SPSS 22.0 (International Business Machines Corp, Armonk, NY, USA).

## Results

Fifteen patients were treated with crizotinib as first line *ALK* inhibition and were followed with^18^F-FDG PET/CT, thirteen had baseline imaging, all had follow up imaging. Median duration of follow up was 11 (2–39) months. Median age of the patients was 57 (21–68) year with 12 females and 3 males. Patient characteristics are given in Table 1. Histology results and ALK status either by FISH and/or immunohistochemistry is given in Table 2.

**Table 1:** Baseline patient characteristics.

	N = 15*
Median age (range)	57 (21–68)
Male/female	3/12
Line of treatment	
First	3
Second	2
Third	4
Fourth	4
Number of patients with metastases in different organs detected by PET/CT before start of crizotinib treatment (N = 13)	
Pulmonary	12**
Mediastinal	13
Hepatic	8
Bone	12
Brain	3
Median PFS during crizotinib treatment in months (N = 15)	6.93 (0.9–26.1)

*2 patients had no FDG-PET/CT baseline imaging study.

** in 1 patient with no pulmonary metastases, the pulmonary metastases became visible within 2 months of therapy.

**Table 2:** Baseline ^18^F-FDG PET and CT tumor response measurements with PERCIST and EORTC criteria and progression-free survival per patient with ALK positive NSCLC.

Patient	Histology	FISH	IHC	CT	FDG PET		PFS
				RECIST	PERCIST	EORTC	
1	Adeno	+	+	PR	PMR -38, 8	PMR	>26.1
2	Adeno	-	+	PR	PMR -42, 6	PMR	4.9
3	Adeno	+	+	PR	PMR -57, 9	PMR	8.3
4	Adeno	+	+	PR	PMR -77, 6	PMR	15.7
5	Adeno	+	nd	PR	PMR -66, 9	PMR	7.8
6	Adeno	+	+	PR	PMR -70, 6	PMR	10.4
7	Adeno	+	+	PR	PMR -76, 6	PMR	9.2
8	Adeno	+	+	PR	PMR -42, 6	PMR	6.9
9	Adeno	+	-	PD	SMD -1, 8	SMD	1.8
10	NSCLC NOS	+	-	PD	PMD 7, 4	PMD	0.9
11	Adeno	+	+	PR	nd	PMR	1.8
12	Adeno	+	+	SD	nd	PMR	6.3
13	Adeno	+	-	PD	nd	PMD	2

Histology is according to 2011 IASLC/ATS/ERS NSCLC classification[12].

Immunohistochemistry (IHC) was performed according to the Ventana ALK scoring

CT response is defined according to RECIST 1.1 criteria.

FDG response is defined according to PERCIST criteria and

1999 EORTC recommendations.

Response in PERCIST criteria is response category with percentage change, weeks after start of therapy.

PR = partial response

SD = stable disease

PD = progressive disease

PMR = partial metabolic response

SMD = stable metabolic disease

PMD = progressive metabolic disease.

PFS = progression-free survival in months.

nd = not determined

### Baseline and 6 weeks CT and ^18^F-FDG PET/CT measured responses

In 13 patients PET/CT was performed during crizotinib treatment. With CT according to RECIST criteria, there were 9 patients with a partial response, 1 with stable disease and 3 patients had progressive disease after 6 weeks of therapy. Median PFS was 6.9 (range 0.9–26.1) months.

**Fig 1:**
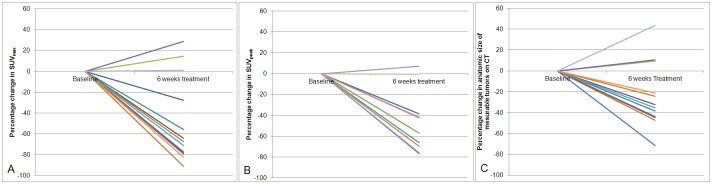
Change in percentage between baseline and after 6 weeks of treatment with crizotinib assessed using SUV_max_ (1A, N = 13), SUV_peak_ (1B, N = 10) and RECIST (1C, N = 13).

**Fig 2:**
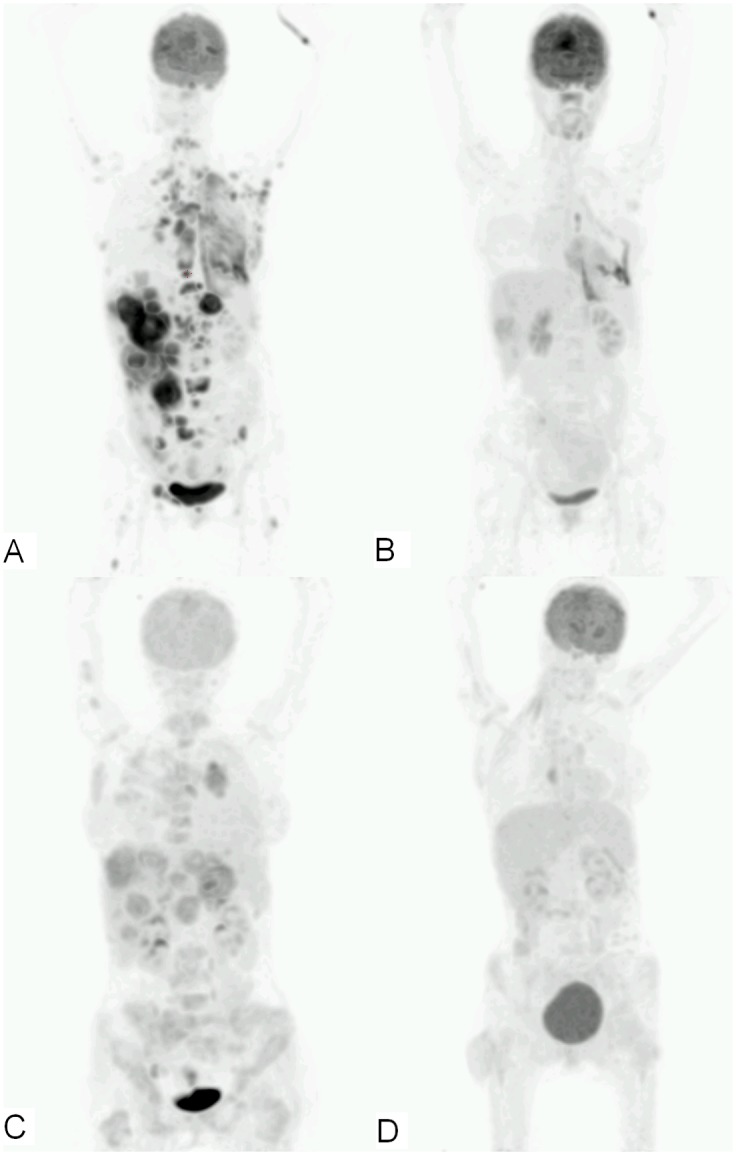
^18^F-FDG maximum intensity projection of patient 2 and 8 prior to (A, B) and after 6 weeks of treatment with crizotinib (C, D). Scale is from 0–15 SUV. These images illustrate the clinically dramatic decrease in ^18^F-FDG uptake, with both patients having a PMR according to both PERCIST criteria and the EORTC recommendations.

With PET measurements according to 1999 EORTC recommendations, there were 10 partial metabolic responders, 1 stabile metabolic disease and 2 progressive metabolic disease. Using the PERCIST criteria in 10 patients, 8 had a partial metabolic response, 1 stabile metabolic disease and 1 progressive metabolic disease (Table 2). There were 2 discordant responses between PET and CT, with a more favorable response on PET (i.e. PMR with SD, or SMD with PD). The per patient change in percentage of SUV_max_ was more pronounced than measured with SUV_peak_ (Fig 1). Although the average outcome using the EORTC recommendations or PERCIST criteria were not impressive, all 10 patients had a clinically dramatic response on the 6 week PET/CT with visual assessment (Fig 2).

Overall, there was a good agreement in the applicability between CT and FDG-PET/CT assessed with EORTC recommendations (N = 13, P = 0.37) at 6 weeks.

### Follow-up with CT and ^18^F-FDG PET/CT

Fifteen patients were included for follow-up, in which additional PET/CT scans were performed. A total of 78 PET/CT were available for evaluation. In 8 out of 15 patients, local progression was detected. Local oligometastatic progression was treated with radio frequency ablation (RFA) in 1 patient, with surgery in 3 patients, and with (stereotactic) radiation in 5 patients. In 6 patients with systemic progression, 4 were treated with ceritinib, 1 with alectinib, and 1 was treated with pemetrexed before receiving ceritinib treatment.

PET/CT was used to detect an increase in metabolic activity at places with previous solid tumors on CT or new lesions that were very small or not yet visible on CT. Comparison of PET and CT according to EORTC criteria at first, second, third line of *ALK* targeted treatment (either systemic or localized treatment) revealed in 5/12, 3/7, 2/3 patients that progressive disease was detected earlier on PET compared with CT. Under first and fourth line treatment one and two patients, respectively, showed no disease progression. This means that in 10/22 (45%) events of progressive disease PET was superior compared to CT. Compared with all assessments, in 10 out of 78 PET/CT, PET alone provided evidence of progression, whereas in 12 out of 78, PET/CT and CT both provided evidence of progression at the same time point.

## Discussion

In this exploratory study the metabolic activity in the primary tumor and metastases decreased dramatically soon after starting crizotinib. There was a good agreement in the applicability between CT and PET based response assessment at 6 weeks. However the metabolic activity decreased to a larger extent than the corresponding tumor size on CT. This result was in line with the good agreement between the measurements according to EORTC recommendations and those measured with PERCIST criteria. The SUV_max_ changes showed the largest absolute decrease in activity. To the best of our knowledge, this is also the first study to compare ^18^F-FDG PET/CT related outcome with ALK immunohistochemistry.

Previously, a study with a murine *ALK* positive NSCLC model in which the *ALK* kinase inhibitor TAE684 was administered, a substantially diminished tumor metabolic activity was detected within 24 hours of starting therapy[18]. One clinical study showed that *ALK* positive NSCLC patients had a higher SUV_max_ than *ALK* negative NSCLC patients, but this difference disappeared in larger tumors[19].

Crizotinib treatment is clearly superior to chemotherapy in treating ALK positive NSCLC patients, with a PFS of 7.7 months[9] yet unfortunately, treatment with targeted therapy commonly leads to acquired resistance. To overcome crizotinib resistance, different therapeutic strategies have been developed [20]. Identifying resistance to treatment at an early moment in individual patients is important, because in solitary or oligometastases localized treatment options such as stereotactic radiotherapy, video-assisted resections or radiofrequency ablation can be applied. Response assessment with ^18^F-FDG PET/CT could represent a method with the ability to identify early resistance to treatment and to identify patients with solitary, oligo or “systemic” metastases. Future research should focus on whether such strategy will improve survival, quality of life and cost-effectiveness. What time point is the best to evaluate an early tumor response? We performed the assessments at 6 weeks but that time point may be too late. At that time there was no difference in test performance between PET and CT. In other targeted treatment modalities with advanced NSCLC, early responses on PET preceded anatomic tumor size alterations[4, 6]. A recent study with surgical resections showed that response monitoring with ^18^F-FDG PET within 1 week of starting treatment with erlotinib in an unselected NSCLC population identified 64% of histopathological responders[21]. The same study also showed that a decrease in ^18^F-FDG activity seen after 1 week of therapy is likely to continue after 3 weeks.

Assessing tumor responses at follow up was easier with PET/CT than with CT. In 10/22 events of disease progression in 15 patients, PET was capable of detecting progression earlier than CT. An additional advantage of PET is that progression is detectable outside of the field of view of a CT. These advantages should be taken into account in cost-effectiveness studies using ^18^F-FDG PET/CT in response assessment during follow-up of oligometastasis.

One problem we encountered, is the discordance between the dramatic results on visual clinical assessment and the less dramatic results using SUV_max_ and SUV_peak_. The weakness of the traditional PET based measurement assessment are based upon the lesion with single highest uptake value, or as we did, with 5 lesions with the highest SUV_max_. It does not take into account the sometimes dramatic decrease of all lesions. Furthermore, it does not take into account lesions that become metabolically inactive. Both response assessment techniques determine progression, with either the appearance of a new lesion or the increased uptake of one lesion to at least a certain percentage compared to previous PET scans. Importantly, the comparison in increased uptake is between the two highest measurable lesions, which does not necessarily need to be the same lesion. An example of the discordance can be described in this example: a patient has 3 lesions. After 6 weeks of treatment the main tumor has a SUV_max_ of 5, the liver lesion a SUV_max_ of 2 and a bone lesion with a SUV_max_ of 3. At the next response scan after 12 weeks of treatment, in the main tumor SUV_max_ decreased to 4, the liver lesion SUV_max_ increased to 6 and the bone lesion remains 3. Because the highest SUV_max_ of the lesions is originally 5 and at the last assessment 6, according to the EORTC recommendations and PERCIST criteria, the patient is not progressive, yet the liver lesion has a clear threefold increase in uptake and clinically the patient has progressive disease. Such a patient is eligible for other forms of targeted therapy and/or a local treatment such as surgery or RFA. With new targeted therapy such as crizotinib, the need to identify examples as the above from patients with systemic disease will become more necessary. It is therefore imperative to reconsider our response criteria as is done for immunotherapy.

## Conclusion

This explorative study of ^18^F-FDG PET/CT in *ALK* positive NSCLC patients treated with crizotinib showed a good agreement between CT and PET measurements at 6 weeks. However, follow up with PET increases early detection of metastases. In 45% of detection of progressive disease events in 15 patients treated with *ALK* inhibitors, PET detected progression of disease earlier than CT did.
